# Prognostic value of serum alkaline phosphatase in spinal metastatic disease

**DOI:** 10.1038/s41416-019-0407-8

**Published:** 2019-02-22

**Authors:** Aditya V. Karhade, Quirina C. B. S. Thio, Megna Kuverji, Paul T. Ogink, Marco L. Ferrone, Joseph H. Schwab

**Affiliations:** 1 Department of Orthopedic Surgery, Massachusetts General Hospital, Harvard Medical School, Boston, MA USA; 2 Department of Orthopedic Surgery, Brigham and Women’s Hospital, Harvard Medical School, Boston, MA USA

**Keywords:** Prognostic markers, Bone metastases

## Abstract

**Background:**

Determination of the appropriateness of invasive management in patients with spinal metastatic disease requires accurate pre-operative estimation of survival. The purpose of this study was to examine serum alkaline phosphatase as a prognostic marker in spinal metastatic disease.

**Methods:**

Chart reviews from two tertiary care centres were used to identify spinal metastatic disease patients. Bivariate and multivariate analyses were used to determine if serum alkaline phosphatase was an independent prognostic marker for survival.

**Results:**

Overall, 732 patients were included with 90-day and 1-year survival of *n* = 539 (74.9%) and *n* = 324 (45.7%), respectively. The 1-year survival of patients in the first quartile of alkaline phosphatase (≤73 IU/L) was 78 (57.8%) compared to 31 (24.0%) for patients in the fourth quartile (>140 IU/L). Preoperative serum alkaline phosphatase levels were significantly elevated in patients with multiple spine metastases, non-spine bone metastasis, and visceral metastasis but not in patients with brain metastasis. On multivariate analysis, elevated serum alkaline phosphatase was identified as an independent prognostic factor for survival in spinal metastatic disease.

**Conclusion:**

Serum alkaline phosphatase is associated with preoperative metastatic tumour burden and is a biomarker for overall survival in spinal metastatic disease.

## Background

Spinal metastases are the most common type of bone metastasis and have a prevalence of 30–50% in cancer patients.^1–3^ Spinal metastases lead to spinal instability, pathologic fractures, neurologic deficits, and decreased quality of life.^1,2^ Management of spinal metastases is primarily palliative and includes consideration of surgery, radiotherapy, medical management, and palliative therapy.^4^ Determination of the appropriateness of invasive management such as multi-level decompression and stabilisation requires accurate pre-operative estimation of survival.^4,5^ A number of prognostic factors have been identified in this population but routinely collected laboratory markers have yet to be fully understood or utilised.^5–7^ Serum alkaline phosphatase is one such marker that is routinely collected in spinal metastatic disease patients but remains underutilised for prognostication.

Alkaline phosphatase has been well-established as a marker of hepatobiliary pathology and bone turnover and mineralisation.^8–11^ This metalloenzyme is expressed on the cell surface of osteoblasts and serum levels of the enzyme correlate with increased osteoblastic activity.^10,12^ In osteolytic bone metastases this enzyme is elevated secondary to a local bone formation response in an attempt to compensate for the predominant destructive lesion.^10^ In osteoblastic bone metastases, alkaline phosphatase is elevated secondary to local stimulation of osteoblasts.^10^ In visceral metastasis, serum alkaline phosphatase is elevated secondary to intrahepatic biliary tract obstruction by hepatic metastatic tumour burden.^11^ On the basis of this known pathophysiology, we hypothesised that elevated serum alkaline phosphatase would be a marker for survival in spinal metastatic disease as an aggregate measure for metastatic tumour burden.

As such, the primary purpose of this study was to determine if serum alkaline phosphatase was an independent prognostic factor for survival in spinal metastatic disease. The secondary aim of this study was to characterise the relationship between serum alkaline phosphatase and metastatic tumour burden.

## Materials and methods

### Guidelines

This study followed the Reporting Recommendations for Tumour Marker Prognostic studies (REMARK) guidelines and the Transparent Reporting of a Multivariable Prediction Model for Individual Prognosis or Diagnosis (TRIPOD) guidelines.^13,14^

### Data source

Chart review was conducted at two tertiary care centres to identify patients with a diagnosis of secondary malignant neoplasm of bone or pathological fracture in metastatic disease. Clinical records for these patients were retrospectively reviewed to ascertain the following inclusion criteria: (1) age >18 years, (2) diagnosis of spinal metastatic disease, (3) initial decompression or stabilisation between 1 January 2000 and 31 December 2016. This study was approved by our institutional review board.

### Outcomes

Post-operative all-cause mortality was the primary outcome in this study. The Social Security Death index was available up to 2014 and was used to establish mortality for this time-period and chart review was used to ascertain mortality thereafter. Ninety-day mortality could be ascertained in 720 (98.4%) of patients and 1-year mortality could be ascertained in 709 (96.9%) of patients.

### Variables

The following pre-operative variables were collected by chart review: age, sex, body mass index [kilograms per metre squared (kg/m^2^)], preoperative presence of any Charlson comorbidity other than metastatic disease,^15^ primary tumour histology [based on slow, moderate, and rapid growth groupings as classified by Katagiri et al.]^16^ (see Appendix Supplementary Table 1 for histology included in each group). Additional factors were pathological fracture at presentation, pain at presentation, Eastern Cooperative Oncology Group performance status, American Spinal Injury Association Impairment Scale, spine tumour location, number of spinal metastases, other non-spine bone metastases, presence of visceral metastases (metastases in liver or lung), presence of brain metastases, history of local radiation to affected site, history of previous systemic therapy, preoperative serum laboratory characteristics in 30-days before surgery: white blood cell [10^3^/microlitre (μL)], haemoglobin [grams per decilitre (g/dL)], platelet (10^3^/μL), absolute lymphocyte (10^3^/μL), absolute neutrophil (10^3^/μL), platelet to lymphocyte ratio, neutrophil to lymphocyte ratio, albumin (g/dL), alkaline phosphatase [international units per litre (IU/L), calcium [milligrams per decilitre (mg/dL)], and creatinine (mg/dL). Operative factors assessed were: number of vertebral levels, surgical approach, surgical type (decompression, stabilisation, corpectomy).

### Missing data

Multiple imputation with Stekhoven et al.’s nonparametric missForest methodology, based on the machine learning methodology of random forests, was used to impute variables with less than 30% missing data.^17^

### Statistical analysis

Descriptive statistics were generated for the baseline characteristics of the population. Bivariate analysis with the non-parametric Mann–Whitney-Wilcoxon test was used to assess the relationship between serum alkaline phosphatase and preoperative metastatic tumour burden (multiple spine metastases, other non-spine bone metastases, visceral metastases, and brain metastases). López-Ratón et al.’s optimal cutpoint method with the area under the receiver operating curve metric was used to determine the threshold for elevated serum alkaline phosphatase.^18^ Kaplan Meier curves were generated for survival by strata of serum alkaline phosphatase. Bivariate Cox proportional hazards analysis was used to assess the association of baseline characteristics with overall survival. Multivariable Cox proportional hazards analysis with bootstrapped (100 replications with replacement) backward stepwise elimination was used to determine if alkaline phosphatase was an independent prognostic factor for overall survival. Sensitivity analyses were conducted by repeating the multivariable Cox proportional hazards analysis with inclusion of alkaline phosphatase as a continuous variable and alkaline phosphatase at other thresholds previously studied in the literature (113, 135 IU/L). Finally, multivariable logistic regression with bootstrapped (100 replications with replacement) backward stepwise elimination was used to determine whether serum alkaline phosphatase was an independent risk factor for mortality at both 90-days and 1-year after surgery.^19,20^

## Results

Of seven hundred and thirty-two spinal metastatic disease patients included in this study, the median age was 61 (interquartile range 53–69) and 206 (41.8%) were female (Table 1). The 30-day, 90-day, and 1-year survival for these patients were *n* = 662 (91.4%), *n* = 539 (74.9%), and *n* = 324 (45.7%), respectively.

**Table 1:** Baseline characteristics of study population, *n* = 732

Variable	*n* (%)
Age (years)	
<65	448 (61.2)
>=65	284 (38.8)
Sex	
Female	306 (41.8)
Male	426 (58.2)
BMI (kg/m^2^)	
18–30	471 (64.3)
<18	21 (2.9)
>30	150 (20.5)
Not recorded	90 (12.3)
Other Charlson comorbidity	441 (60.2)
Primary Tumour Histology	
Group 1 (slow growth)	219 (29.9)
Group 2 (moderate growth)	254 (34.7)
Group 3 (rapid growth)	259 (35.4)
Pathological fracture	456 (62.3)
Pain	627 (85.7)
ECOG	
0–2	440 (60.1)
3–4	99 (13.5)
Not recorded	193 (26.4)
ASIA	
Normal (E)	379 (51.8)
Impaired (A–D)	342 (46.7)
Not recorded	11 (1.5)
Tumour location	
Cervical	104 (14.2)
Thoracic	425 (58.1)
Lumbar	164 (22.4)
Multiple	39 (5.3)
Spine metastases	
One	211 (28.8)
Two	117 (16.0)
Three or more	404 (55.2)
Other bone metastases	388 (53.0)
Visceral metastases	252 (34.4)
Brain metastases	81 (11.1)
History of local radiation	252 (34.4)
Previous systemic therapy	418 (57.1)
White blood cell (10^3^/μL)	
<11	486 (66.4)
>=11	157 (21.4)
Not measured	89 (12.2)
Haemoglobin (g/dL)	
<13	435 (59.4)
>=13	208 (28.4)
Not measured	89 (12.2)
Platelet (10^3^/μL)	
<150	75 (10.2)
>450	50 (6.8)
150–450	518 (70.8)
Not measured	89 (12.2)
Absolute lymphocyte (10^3^/μL)	
<1	280 (38.3)
>=1	234 (32.0)
Not measured	218 (29.8)
Absolute neutrophil (10^3^/μL)	
<6	232 (31.7)
>=6	285 (38.9)
Not measured	215 (29.4)
Neutrophil to lymphocyte ratio	
<4.7	174 (23.8)
>=4.7	340 (46.4)
Not measured	218 (29.8)
Platelet to lymphocyte ratio	
<408	355 (48.5)
>=408	159 (21.7)
Not measured	218 (29.8)
Albumin (g/dL)	
<3.5	156 (21.3)
>=3.5	391 (53.4)
Not measured	185 (25.3)
Alkaline phosphatase (IU/L)	
<100	290 (39.6)
>=100	248 (33.9)
Not measured	194 (26.5)
Calcium (mg/dL)	
<9	257 (35.1)
>=9	365 (49.9)
Not measured	110 (15.0)
Creatinine (mg/dL)	
<1	467 (63.8)
>=1	176 (24.0)
Not measured	89 (12.2)
Number of levels operated	
One or two	531 (72.5)
Three or more	200 (27.3)
Anterior approach	105 (14.3)
Posterior approach	660 (90.2)
Combined approach	33 (4.5)
Decompression	699 (95.5)
Stabilisation	640 (87.4)
Corpectomy	351 (48.0)

*ASIA* American Spinal Injury Association Impairment Scale, *BMI* body mass index, *ECOG* Eastern Cooperative Oncology Group performance status, (*g/dL*) grams per decilitre, (*IU/L*) international units per litre, (*kg/m*^2^) kilogram per metre squared, (*mg/dL*) milligrams per decilitre, *μL* microlitre

**Fig. 1:**
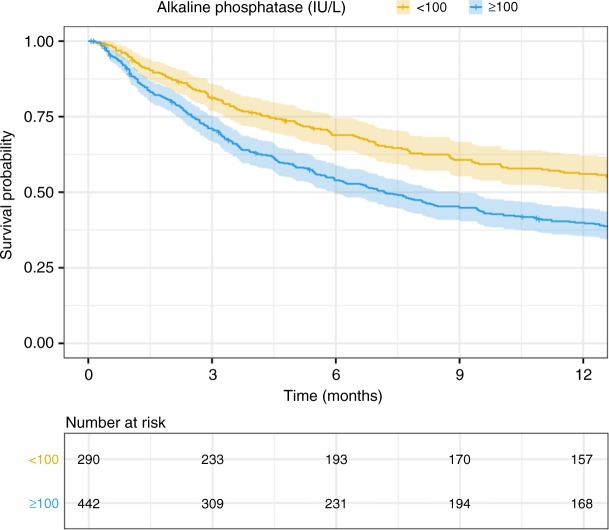
Kaplan–Meier curve by strata of serum alkaline phosphatase. Patients with elevated preoperative serum alkaline phosphatase had lower predicted survival at all time points up to 1-year

The optimal cut-off for serum alkaline phosphatase was determined to be 100 international units per litre (IU/L) using the area under the receiver operating curve metric. Patients with alkaline phosphatase ≥100 IU/L had shorter predicted postoperative survival at all time points up to 1-year (Fig. 1). The 1-year survival by quartile of serum alkaline phosphatase was: 78 (57.8%) for alkaline phosphatase ≤73 IU/L, 71 (55.0%) for 73 IU/L < alkaline phosphatase ≤94.5 IU/L, 50 (37.9%) for 94.5 IU/L < alkaline phosphatase ≤140 IU/L, and 31 (24.0%) for alkaline phosphatase >140 IU/L.

### Metastatic tumour burden

**Fig. 2:**
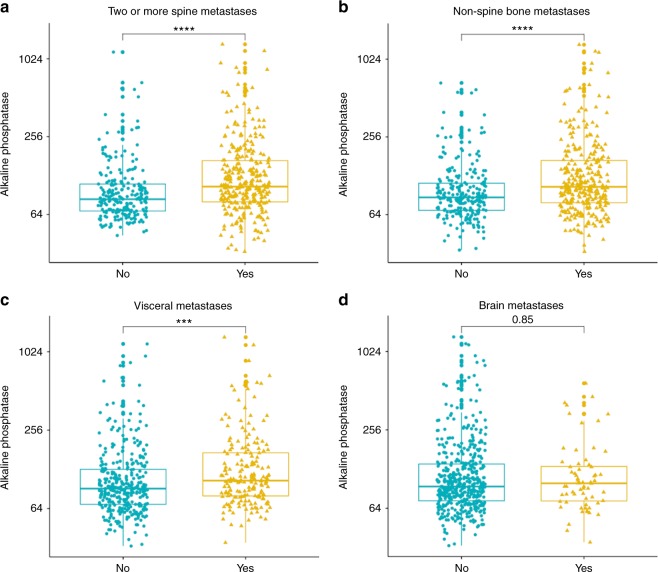
**a**–**c** Serum alkaline phosphatase was associated with preoperative burden of multiple spine metastases, non-spine metastases, and visceral metastases (***p < 0.001). **d** Serum alkaline phosphatase was not associated with preoperative burden of brain metastases (*p* = 0.85)

In patients with multiple spine metastasis, *n* = 521 (71.2%), serum alkaline phosphatase was, median (interquartile range), 102 (76–154) IU/L whereas in patients with a single spine metastasis, serum alkaline phosphatase was 84 (69–106) IU/L (*p* < 0.001). In patients with other non-spine bone metastasis, *n* = 388 (53%), serum alkaline phosphatase was 105 (79–168) IU/L whereas in patients with no non-spine bone metastasis, serum alkaline phosphatase levels were 87 (69–112) IU/L (*p* < 0.001). In patients with visceral metastasis, *n* = 252 (34.4%), serum alkaline phosphatase was 105 (80–172) IU/L whereas in patients with no visceral metastasis, serum alkaline phosphatase levels were 91 (69–128) IU/L (*p* < 0.001). In patients with brain metastasis, *n* = 81 (11.1%), serum alkaline phosphatase was 100 (73–134) IU/L whereas in patients with no brain metastasis, serum alkaline phosphatase levels were 94 (73–140) IU/L (*p* = 0.85). Overall, serum alkaline phosphatase levels were significantly elevated in patients with multiple spine metastases (*p* < 0.001), non-spine bone metastasis (*p* < 0.001), and visceral metastasis (*p* < 0.001) but not in patients with brain metastasis (*p* = 0.85) (Fig. 2).

### Survival analysis

On bivariate Cox proportional hazards analysis, primary tumour histology, BMI, concurrent medical comorbidities, performance status, neurologic deficit, spinal location, metastatic tumour burden (multiple spine metastases, other non-spine bone metastases, visceral metastases, brain metastases), history of local radiation, history of previous systemic therapy, anaemia, thrombocytopenia, thrombocytosis, lymphocytopenia, neutrophilia, NLR, PLR, hypoalbuminemia, alkaline phosphatase, calcium, creatinine, and more invasive surgery were associated with survival (Table 2).

**Table 2:** Bivariate Cox proportional hazards regression analyses of baseline characteristics, *n* = 732

Variable	Odds ratio	95% CI	*p*-value
Age (years)	1.07	(0.90, 1.26)	0.45
Female sex	1.00	(0.85, 1.18)	0.98
BMI (kg/m^2^)			
18–30	*Reference*	*–*	*–*
<18	1.83	(1.14, 2.95)	0.01
>30	0.85	(0.69, 1.04)	0.12
Other Charlson comorbidity	1.26	(1.06, 1.49)	0.007
Primary Tumour Histology			
Group 1 (slow growth)	*Reference*	*–*	*–*
Group 2 (moderate growth)	1.74	(1.40, 2.15)	<0.001
Group 3 (rapid growth)	3.21	(2.60, 3.98)	<0.001
Pathological fracture	1.13	(0.96, 1.34)	0.14
Pain	0.97	(0.78, 1.22)	0.8
ECOG 3–4	2.65	(2.10, 3.35)	<0.001
ASIA Impaired (A-D)	1.5	(1.28, 1.77)	<0.001
Cervical Metastasis	1.09	(0.86, 1.38)	0.47
Thoracic Metastasis	1.21	(1.02, 1.43)	0.03
Lumbar Metastasis	0.74	(0.61, 0.91)	0.004
Two or More Spine Metastases	1.64	(1.36, 1.98)	<0.001
Other Bone Metastases	1.43	(1.21, 1.68)	<0.001
Visceral Metastases	1.77	(1.49, 2.10)	<0.001
Brain Metastases	2.07	(1.63, 2.63)	<0.001
History of local radiation	1.23	(1.04, 1.46)	0.02
Previous systemic therapy	1.78	(1.50, 2.11)	<0.001
White Blood Cell (10^3^/μL) > = 11	1.15	(0.94, 1.40)	0.17
Haemoglobin (g/dL) < 13	1.68	(1.39, 2.04)	<0.001
Platelet (×10^3^/μL)			
150–450	*Reference*	*–*	*–*
<150	1.47	(1.13, 1.92)	0.004
>450	1.44	(1.05, 1.97)	0.02
Absolute lymphocyte (10^3^/μL) < 1	1.60	(1.32, 1.94)	<0.001
Absolute neutrophil (10^3^/μL) > 6	1.28	(1.05, 1.55)	0.01
Neutrophil to lymphocyte ratio >=4.7	1.80	(1.46, 2.22)	<0.001
Platelet to lymphocyte ratio >=408	1.66	(1.36, 2.04)	<0.001
Albumin (g/dL) < 3.5	1.97	(1.61, 2.41)	<0.001
**Alkaline phosphatase (IU/L)** **>=** **100**	**1.84**	**(1.52, 2.22)**	**<0.001**
Calcium (mg/dL) >= 9	0.73	(0.62, 0.88)	<0.001
Creatinine (mg/dL) >= 1	0.77	(0.64, 0.94)	0.009
Number of levels operated	1.16	(0.96, 1.40)	0.12
Anterior approach	0.75	(0.60, 0.95)	0.02
Posterior approach	1.12	(0.86, 1.47)	0.41
Combined approach	0.57	(0.37, 0.87)	0.01
Decompression	1.23	(0.81, 1.86)	0.34
Stabilisation	0.74	(0.59, 0.94)	0.01
Corpectomy	0.86	(0.73, 1.02)	0.08

*ASIA* American Spinal Injury Association Impairment Scale, *BMI* body mass index, *ECOG* Eastern Cooperative Oncology Group performance status, (*g/dL*) grams per decilitre, (*IU/L*) international units per litre, (*mg/dL*) milligrams per decilitre, *μL* microlitre

Serum alkaline phosphatase provided in bold

On multivariate Cox proportional hazards analysis, serum alkaline phosphatase > 100 IU/L remained an independent prognostic factor for overall survival (Table 3). On sensitivity analyses with assessment of serum alkaline phosphatase as a continuous variable and alkaline phosphatase at thresholds of 113 IU/L and 135 IU/L, serum alkaline phosphatase remained an independent prognostic factor for survival [Supplementary Tables 2–4].

**Table 3:** Multivariate Cox-proportional hazards regression, *n* = 732

Variable	HR	95 % CI	*p*-value
Other Charlson comorbidity	1.21	(1.02, 1.43)	0.03
Primary Tumour Histology			
Group 1 (slow growth)	*Reference*	*–*	*–*
Group 2 (moderate growth)	1.55	(1.25, 1.94)	<0.001
Group 3 (rapid growth)	2.99	(2.38, 3.75)	<0.001
ECOG 3–4	2.66	(2.14, 3.31)	<0.001
Two or more spine metastases	1.36	(1.11, 1.65)	0.002
Other bone metastases	1.24	(1.04, 1.49)	0.02
Visceral metastases	1.17	(0.97, 1.41)	0.09
Brain metastases	1.43	(1.12, 1.83)	0.004
Previous systemic therapy	1.42	(1.19, 1.7)	<0.001
Hemoglobin (g/dL) < 13	1.38	(1.13, 1.67)	0.001
Platelet to lymphocyte ratio >= 408	1.17	(0.98, 1.41)	0.08
Albumin (g/dL) < 3.5	2.03	(1.68, 2.46)	<0.001
Alkaline Phosphatase (IU/L) >= 100	1.28	(1.07, 1.52)	0.006

*ECOG* Eastern Cooperative Oncology Group performance status, (*g/dL*) grams per decilitre, (*IU/L*) international units per litre

On multivariable logistic regression of 90-day mortality, serum alkaline phosphatase (continuous) was an independent prognostic factor [Supplementary Table 5]. On multivariable logistic regression of 1-year mortality, serum alkaline phosphatase (continuous) was an independent prognostic factor [Supplementary Table 6].

## Discussion

In this population of 732 patients undergoing intervention for spinal metastatic disease, serum alkaline phosphatase was significantly elevated in patients with multiple spinal metastases, other non-spine bone metastases, and visceral metastases but not in patients with brain metastases. Elevated serum alkaline phosphatase was identified as an independent prognostic factor for survival on multivariate Cox proportional hazard analyses and further confirmed as a prognostic factor at both 90-days and 1-year after surgery on multivariate logistic regression analyses.

In metastatic disease, alkaline phosphatase has been previously examined as a diagnostic marker for the presence of liver and bone metastases. Tartter et al.^21^ retrospectively studied 327 patients with colorectal cancer and found sensitivity of 88% and false positive rate of 12% for the presence of liver metastases with serum alkaline phosphatase >135 IU/L and carcinoembryonic antigen greater than 10 ng/ml. Seamen et al.^22^ studied 90 patients with metastatic renal cell cancer and found that the presence of elevated alkaline phosphatase (>100 IU/L) and/or bone pain was able to identify 27 of 28 patients with bone metastases identified on radionucleotide bone scan. In this study, the finding of elevated alkaline phosphatase in patients with multiple spine metastases, non-spine metastasis, and visceral metastasis supports both the pathophysiology of alkaline phosphatase and the findings of previous studies. The additional finding of no significant association with the presence of brain metastases served as an important control as the elevation of alkaline phosphatase was specific to bone and visceral metastases.

Previous studies have also examined serum alkaline phosphatase as a prognostic marker in malignancy. Gu et al.^23^ conducted a meta-analysis of eleven osteosarcoma studies between 1993 and 2013 with 1336 patients and found stable pooled hazard ratios confirming that elevated serum alkaline phosphatase was associated with poor survival. Manola et al.^24^ conducted a pooled 25-year analysis of 1362 metastatic melanoma patients from eight Eastern Cooperative Oncology Group trials and identified alkaline phosphatase as predictive of poor survival on proportional hazards modelling. Berry et al., Emrich et al. and Kantoff et al. identified serum alkaline phosphatase as a prognostic biomarker in metastatic prostate cancer and Halabi et al. developed a nomogram for survival probability in metastatic hormone-refractory prostate cancer including serum alkaline phosphatase.^25–28^ Serum alkaline phosphatase was recently studied as risk factor for 30-day postoperative mortality in spinal metastatic disease patients in the National Surgical Quality Improvement Program (NSQIP), a database of short-term postoperative outcomes in North America.^20^ This database is fairly limited for oncologic studies because it lacks follow-up beyond 30-days and does not include important prognostic factors such as primary tumour histology, history of radiation, history of systemic therapy, visceral metastases, previous systemic therapy or history of local radiation.^29^ The findings of the present study extend the findings of prior studies of alkaline phosphatase in metastatic disease and establish a role for short and long-term prognostication in spinal metastatic disease.

Previous prognostic studies of spinal metastatic disease patients have identified preoperative haemoglobin,^30^ white blood cell count,^30^ absolute neutrophil-to-lymphocyte ratio,^31^ platelet-to-lymphocyte ratio,^31^ calcium,^32^ and preoperative albumin^32–34^ as laboratory markers for postoperative mortality and morbidity. In this population of spinal metastatic disease patients, serum alkaline phosphatase was collected as part of routine liver function tests in greater than 70% of patients in the 30-days before surgery. This existing collection of serum alkaline phosphatase but underutilisation for prognostication highlights an opportunity for the findings of this study to suggest new avenues for more efficiently using existing healthcare resources and improving the value of care delivery in metastatic disease.

Furthermore, well established scoring systems such as the Bauer,^35^ New England Spinal Metatasis,^36^ Katagiri,^37^ Sioutos,^7^ SORG,^38^ Tokuhashi,^39^ Tomita,^40^ van der Linden^41^ and others^6,42^ currently incorporate measures of preoperative metastatic tumour burden by assessing the number of spine metastasis, other non-spine bone metastasis and visceral metastasis. However, despite controlling for these and other factors, serum alkaline phosphatase remained an independent prognostic factor on multivariable analysis; in addition, visceral metastasis no longer reached significance after incorporation of serum alkaline phosphatase. This suggests that existing scoring systems in spinal metastatic disease should be updated by additionally considering preoperative serum alkaline phosphatase as a candidate predictor.

There are several limitations to this study. The patient population was drawn from a single region and two tertiary care centres. Additionally, this study was retrospective and prospective validation of serum alkaline phosphatase as independent prognostic factor remains to be undertaken. All patients in this study underwent initial definitive surgical intervention for metastatic disease and the role of alkaline phosphatase in spinal metastatic disease managed with only radiotherapy, chemotherapy or palliative care remains to be determined. In addition, anti-resorptive therapy in metastatic bone disease decreases adverse skeletal-related events, such as pathologic fractures and spinal cord compression, and is reflected by reductions in biomarkers of bone turnover including alkaline phosphatase.^43,44^ In patients receiving preoperative anti-resorptive therapy, elevated serum alkaline phosphatase may be a marker for missing response to anti-resorptive agents. As such, future studies of spinal metastatic disease patients undergoing operative intervention should further explore preoperative treatment with anti-resorptive therapy in relation to serum alkaline phosphatase levels at presentation as well as the role for postoperative prognostication on the basis of response to this administered therapy.

Nonetheless, this study identified serum alkaline phosphatase as an independent prognostic factor for overall survival in patients undergoing surgery for spinal metastatic disease and provided greater understanding of the relationship between this readily available laboratory marker and preoperative metastatic tumour burden. Future studies should consider assessment of this marker in the creation of nomograms and prediction models for spinal metastatic disease.

## Conclusion

Serum alkaline phosphatase is associated with preoperative metastatic tumour burden (spine, bone, visceral) and is a biomarker for overall survival in spinal metastatic disease. Future studies building prognostic models for spinal metastatic disease should consider assessment of this simple preoperative biomarker.

## Supplementary information

Appendix

## Data Availability

All data generated or analysed during this study are included in this published article. The corresponding author of this study will accept reasonable requests for data that support the findings of this study.
