# Supplementary material for: Prognostic value of serum alkaline phosphatase in spinal metastatic disease
Source: Br J Cancer. 2019 Feb 22;120(6):640–6. doi: 10.1038/s41416-019-0407-8 (PMC6461951; doi:10.1038/s41416-019-0407-8)
Supplement: Supplementary file 1 — Appendix [file 41416_2019_407_MOESM1_ESM.docx]

Appendix:

Supplementary Table 1: Histology groupings, based on Katagiri et al.^12^

| Group | Histology |
| --- | --- |
| Slow growth  (Group 1) | Hormone dependent breast cancer, hormone dependent prostate cancer, malignant lymphoma, malignant myeloma, thyroid cancer |
| Moderate growth  (Group 2) | Non-small cell lung cancer with molecularly targeted therapy, hormone independent breast cancer, hormone independent prostate cancer, renal cell carcinoma, sarcoma, other gynecological cancer, others |
| Rapid growth  (Group 3) | Other lung cancer, colon and rectal cancer, gastric cancer, hepatocellular carcinoma, pancreatic cancer, head and neck cancer, other urological cancer, esophageal cancer, malignant melanoma, gallbladder cancer, cervical cancer, unknown origin. |

Supplementary Table 2: Multivariate Cox-proportional hazards regression with alkaline phosphatase as a continuous marker, n = 732

| **Variable** | **HR** | **95 % CI** | **P-value** |
| --- | --- | --- | --- |
| Other Charlson comorbidity | 1.19 | (1, 1.41) | 0.048 |
| Primary Tumor Histology |  |  |  |
| Group 1 (slow growth) | *Reference* | *-* | *-* |
| Group 2 (moderate growth) | 1.54 | (1.24, 1.93) | <0.001 |
| Group 3 (rapid growth) | 3.05 | (2.43, 3.82) | <0.001 |
| ECOG 3-4 | 2.57 | (2.06, 3.2) | <0.001 |
| Two or More Spine Metastases | 1.33 | (1.09, 1.62) | 0.005 |
| Other Bone Metastases | 1.21 | (1.01, 1.45) | 0.034 |
| Visceral Metastases | 1.18 | (0.99, 1.42) | 0.071 |
| Brain Metastases | 1.44 | (1.13, 1.84) | 0.003 |
| Previous Systemic Therapy | 1.37 | (1.14, 1.64) | 0.001 |
| Hemoglobin (g/dL) < 13 | 1.34 | (1.10, 1.63) | 0.003 |
| Platelet to Lymphocyte Ratio >= 408 | 1.23 | (1.02, 1.47) | 0.028 |
| Albumin (g/dL) < 3.5 | 1.99 | (1.65, 2.40) | <0.001 |
| Alkaline Phosphatase (IU/L) | 1.002 | (1.001, 1.002) | <0.001 |
| *Abbreviations: Eastern Cooperative Oncology Group performance status; (g/dL): grams per deciliter; (IU/L: international units per liter* | | | |

Supplementary Table 3: Multivariate Cox-proportional hazards regression with alkaline phosphatase dichotomized at 113 IU/L, n = 732

| **Variable** | **HR** | **95 % CI** | **P-value** |
| --- | --- | --- | --- |
| Other Charlson comorbidity | 1.20 | (1.01, 1.42) | 0.039 |
| Primary Tumor Histology |  |  |  |
| Group 1 (slow growth) | *Reference* | *-* | *-* |
| Group 2 (moderate growth) | 1.57 | (1.26, 1.95) | <0.001 |
| Group 3 (rapid growth) | 2.96 | (2.36, 3.72) | <0.001 |
| ECOG 3-4 | 2.66 | (2.14, 3.31) | <0.001 |
| Two or More Spine Metastases | 1.34 | (1.10, 1.63) | 0.003 |
| Other Bone Metastases | 1.25 | (1.05, 1.49) | 0.014 |
| Visceral Metastases | 1.20 | (1.00, 1.44) | 0.052 |
| Brain Metastases | 1.44 | (1.12, 1.84) | 0.004 |
| Previous Systemic Therapy | 1.40 | (1.17, 1.68) | <0.001 |
| Hemoglobin (g/dL) < 13 | 1.37 | (1.12, 1.66) | 0.002 |
| Platelet to Lymphocyte Ratio >= 408 | 1.18 | (0.98, 1.41) | 0.079 |
| Albumin (g/dL) < 3.5 | 2.06 | (1.70, 2.50) | <0.001 |
| Alkaline Phosphatase (IU/L) >= 113 | 1.33 | (1.12, 1.58) | 0.001 |
| *Abbreviations: Eastern Cooperative Oncology Group performance status; (g/dL): grams per deciliter; (IU/L): international units per liter* | | | |

Supplementary Table 4: Multivariate Cox-proportional hazards regression with alkaline phosphatase dichotomized at 135 IU/L, n = 732

| **Variable** | **HR** | **95 % CI** | **P-value** |
| --- | --- | --- | --- |
| Other Charlson comorbidity | 1.20 | (1.01, 1.42) | 0.04 |
| Primary Tumor Histology |  |  |  |
| Group 1 (slow growth) | *Reference* | *-* | *-* |
| Group 2 (moderate growth) | 1.55 | (1.25, 1.94) | <0.001 |
| Group 3 (rapid growth) | 2.98 | (2.38, 3.75) | <0.001 |
| ECOG 3-4 | 2.67 | (2.15, 3.33) | <0.001 |
| Two or More Spine Metastases | 1.32 | (1.08, 1.60) | 0.006 |
| Other Bone Metastases | 1.24 | (1.04, 1.49) | 0.017 |
| Visceral Metastases | 1.20 | (1.00, 1.45) | 0.047 |
| Brain Metastases | 1.44 | (1.13, 1.84) | 0.004 |
| Previous Systemic Therapy | 1.39 | (1.16, 1.67) | <0.001 |
| Hemoglobin (g/dL) < 13 | 1.36 | (1.12, 1.66) | 0.002 |
| Platelet to Lymphocyte Ratio >= 408 | 1.16 | (0.97, 1.39) | 0.115 |
| Albumin (g/dL) < 3.5 | 2.01 | (1.67, 2.43) | <0.001 |
| Alkaline Phosphatase (IU/L) >= 135 | 1.34 | (1.12, 1.59) | 0.001 |
| *Abbreviations: Eastern Cooperative Oncology Group performance status; (g/dL): grams per deciliter; (IU/L): international units per liter* | | | |

Supplementary Table 5: Multivariate analysis of ninety-day mortality with serum alkaline phosphatase, n = 732

| **Variable** | **Odds Ratio** | **95% CI** | **P-value** |
| --- | --- | --- | --- |
| Primary Tumor Histology |  |  |  |
| Group 1 | *Reference* | - | - |
| Group 2 | 1.54 | (0.85, 2.80) | 0.15 |
| Group 3 | 4.72 | (2.67, 8.36) | <0.001 |
| ECOG 3-4 | 2.99 | (1.85, 4.85) | <0.001 |
| Visceral Metastases | 1.33 | (0.86, 2.06) | 0.20 |
| Brain Metastases | 1.64 | (0.90, 2.98) | 0.10 |
| Other Bone Metastases | 1.16 | (0.73, 1.82) | 0.52 |
| Two or More Spine Metastases | 1.71 | (1.00, 2.91) | 0.05 |
| Hemoglobin (g/dL) < 13 | 1.38 | (0.82, 2.30) | 0.22 |
| Platelet to Lymphocyte Ratio >= 408 | 1.30 | (0.80, 2.09) | 0.29 |
| Neutrophil to Lymphocyte Ratio >= 4.7 | 2.65 | (1.47, 4.78) | 0.001 |
| Albumin (g/dL) < 3.5 | 5.27 | (3.44, 8.07) | <0.001 |
| Alkaline phosphatase (IU/L – continuous) | 1.002 | (1.001, 1.004) | 0.004 |
| *Abbreviations: ASIA: American Spinal Injury Association Impairment Scale; ECOG: Eastern Cooperative Oncology Group performance status; (g/dL): grams per deciliter; (IU/L): international units per liter* | | | |

Supplementary Table 6: Multivariate analysis of one-year mortality with serum alkaline phosphatase, n = 732

| **Variable** | **Odds Ratio** | **95% CI** | **P-value** |
| --- | --- | --- | --- |
| Other Charlson comorbidity | 1.67 | (1.12, 2.48) | 0.01 |
| Primary Tumor Histology |  |  |  |
| Group 1 | *Reference* | - | - |
| Group 2 | 2.14 | (1.33, 3.47) | 0.002 |
| Group 3 | 10.6 | (6.21, 18.2) | <0.001 |
| Pain | 2.30 | (1.29, 4.10) | 0.005 |
| ECOG | 4.74 | (2.45, 9.20) | <0.001 |
| ASIA | 1.76 | (1.18, 2.64) | 0.006 |
| Visceral Metastases | 1.60 | (1.05, 2.46) | 0.03 |
| Brain Metastases | 1.22 | (0.64, 2.35) | 0.54 |
| Other Bone Metastases | 1.39 | (0.91, 2.13) | 0.13 |
| Two or More Spine Metastases | 1.36 | (0.86, 2.16) | 0.19 |
| Previous Systemic Therapy | 1.89 | (1.26, 2.84) | 0.002 |
| Hemoglobin (g/dL) < 13 | 1.74 | (1.12, 2.71) | 0.01 |
| Albumin (g/dL) < 3.5 | 3.06 | (1.87, 5.02) | <0.001 |
| Alkaline phosphatase (IU/L – continuous) | 1.004 | (1.001, 1.06) | 0.002 |
| Lumbar Metastasis | 0.64 | (0.41, 1.02) | 0.06 |
| Platelet to Lymphocyte Ratio >= 408 | 1.62 | (0.99, 2.66) | 0.06 |
| Neutrophil to Lymphocyte Ratio >= 4.7 | 1.67 | (1.04, 2.68) | 0.03 |
| *Abbreviations: ECOG: Eastern Cooperative Oncology Group performance status; (g/dL): grams per deciliter; (IU/L): international units per liter* | | | |
